# Clinico-pathological features and related risk factors of Type-2 diabetes mellitus complicated with nonalcoholic fatty liver

**DOI:** 10.12669/pjms.38.7.6289

**Published:** 2022

**Authors:** Lisha Chen, Li Jiang

**Affiliations:** 1 Lisha Chen, Department of Infectious Diseases, Wenling First People’s Hospital, Wenling 317500, Zhejiang Province, P.R. China; 2 Li Jiang, Department of Infectious Diseases, Wenling First People’s Hospital, Wenling 317500, Zhejiang Province, P.R. China

**Keywords:** Type-2 diabetes mellitus, Nonalcoholic fatty liver disease, Obesity, Insulin resistance, Lipid metabolism

## Abstract

**Objectives:**

To analyze the clinicopathological features and risk factors of Type-2 diabetes mellitus (T2DM) patients with non-alcoholic fatty liver disease (NAFLD).

**Methods:**

The data of 145 patients with T2DM who received treatment in our hospital from May 2020 to May 2021 were collected. The patients were diagnosed with NAFLD by abdominal liver Doppler ultrasound; The general data and laboratory examination indexes of T2DM patients with and without NAFLD were compared; To analyze the risk factors of NAFLD in T2DM patients.

**Results:**

According to the results of the ultrasound examination, 71(48.97%) patients were simple T2DM, and 74(51.03%) patients were T2DM with NAFLD. Compared with simple T2DM, T2DM patients with NAFLD had higher BMI, hypertension, fasting plasma glucose(FPG), insulin resistance, triglycerides (TG), alanine aminotransferase (ALT), aspartate aminotransferase (AST) and uric acid(UA) (*P*<0.05). Further logistic regression analysis showed a higher BMI (*OR*=1.841, *P*=0.013), FPG (*OR*=1.576, *P*=0.014), insulin resistance (*OR*=4.195, *P*<0.001) and elevated TG (*OR*=4.676, *P*=0.042) are risk factors for T2DM with NAFLD.

**Conclusion:**

High BMI, BPG, insulin resistance index and TG are independent risk factors for nonalcoholic fatty liver in T2DM patients. During intervention, attention should be paid to the monitoring of these indicators to effectively prevent the aggravation of the disease.

## INTRODUCTION

Type-2 diabetes mellitus (T2DM) is a common metabolic disease, affecting over 451 million people worldwide.^1^ The symptoms of T2DM can seriously reduce the quality of life of these patients, leading to complications, such as nonalcoholic fatty liver disease (NAFLD).^2^,^3^ NAFLD can progress to liver fibrosis, induce liver cirrhosis, and may even lead to liver cancer. The prevalence of NAFLD within the general population is only 5.0%, but it is much higher within patients with T2DM, increasing to 25.0~75.0%.^4^,^5^ Further studies have found that about 21 to 45% of NAFLD patients had T2DM, with 30% of them having a family history of diabetes.^6^

T2DM patients complicated with NAFLD will also present with insulin resistance, as the increased fat within the liver directly affects hepatic glucose output, and increases the risk of liver cirrhosis and liver cancer.^7^ Insulin resistance and hyperinsulinemia are the main characteristics of T2DM. The pathogenesis of NAFLD focuses on the “second strike” theory, whereby insulin resistance causes the first strike, resulting in fatty liver lesions. The second strike, due to NAFLD, results from lipid peroxidation, causing inflammatory cell infiltration and hepatocyte necrosis.^8^

Currently, the pathogenesis of T2DM complicated with NAFLD has not been fully clarified and has limited available treatment. The current treatment consists of drug application and lifestyle intervention, with limited overall effects.^9^ Therefore, for T2DM complicated with NAFLD, early identification of risk factors and targeted prevention strategies are of paramount importance. The purpose of this study was to analyze the pathophysiological characteristics and risk factors of T2DM complicated with NAFLD. Both patients with T2DM only and those complicated with NAFLD treated at our hospital from May 2020 to May 2021, were retrospectively compared to further clarify the risk factors of the disease.

## METHODS

The records of T2DM patients treated in our hospital from May 2020 to May 2021 were collected, with a total of 145 cases; 88 males and 57 females; Age 18~70 years old. This study has been approved by the medical ethics association of our hospital (No.:KY-2021-2051-01).

### Inclusion criteria:

- Meeting the diagnostic criteria of T2DM proposed by WHO,^10^ the diagnostic basis of NAFLD is that T2DM has existed in the past. During hospitalization, fasting digestive system color ultrasound was performed in the color ultrasound room of our hospital to indicate fatty liver;^11^
- Complete medical history;

### Exclusion criteria:

- Fatty liver caused by other reasons;
- Other special types of diabetes;
- Pregnant and lactating women.

The gender, age, duration of diabetes, weight, height, history of hypertension, and smoking habits were recorded and body mass index (BMI; BMI=body weight/height^2^) was calculated. Fasting plasma glucose (FPG) was detected by Hitachi 7600 of Hangzhou ruixie Technology Co. Ltd, using the glucose oxidase detection method. C peptide (CP) was detected by electrochemiluminescence. Pass 1.5+FPG after testing×CP/2800 insulin resistance index was calculated.^12^ Total cholesterol (TC), triglyceride (TG), low density lipoprotein (LDL) and high-density lipoprotein (HDL), glycosylated hemoglobin (HbA1c), alanine aminotransferase (ALT) and aspartate aminotransferase (AST) were detected using the electrode method. Creatinine(CR) and uric acid (UA) were detected using an automatic biochemical analyzer (Roche, Olympus au-2700, USA).

The data were processed by Spss22.0, and [n(%)] was used to represent the non-grade count data. The test method was *χ^2^*, and (*X̅*±*S*) was used to represent the measurement data. T-test was used for the parametric and the rank sum test was used for the non-parametric data. The risk factors were analyzed by binary logistic regression and *P*<0.05 was considered statistically significant.

## RESULTS

A total of 145 T2DM patients met the inclusion criteria, including 88 males and 57 females; Age 18~70 years old; There were 71(48.97%) patients with simple T2DM and 74 (51.03%) patients with T2DM and NAFLD. There was no significant difference in gender, age, duration of diabetes or smoking habits between patients with T2DM alone and those T2DM with NAFLD (*P*>0.05). T2DM with NAFLD was associated with higher BMI compared to T2DM alone (BMI of 25.54±0.83 versus 27.08±1.49, respectively, p<0.001), and hypertension (BP ≥140/90mmHg) (*P*<0.05; Table-I). The levels of FPG, insulin resistance, TG, ALT, AST and UA of T2DM with NAFLD were higher than those of T2DM alone (*P*<0.05; Table-II). There was no significant difference in the levels of TC, LDL, HDL, HbAlc and Cr between the two groups (*P*>0.05), as shown in Table-II. BMI (*OR*=1.841, *P*=0.013), FPG (*OR*=1.576, *P*=0.014), insulin resistance index (*OR*=4.195, *P*<0.001) and TG (*OR*=4.676, *P*=0.042) are risk factors for T2DM with NAFLD (Table-III).

**Table I:** Comparison of basic clinical characteristics between patients with T2DM and T2DM with NAFLD [n(%),*X̅*±*S*].

Basic Features	Simple T2DM (n=71)	T2DM with NAFLD (n=74)	χ^2^ t	p-Value
Gender (Male /Female)	43/28	45/29	0.001	0.976
Age (years)	52.22±11.85	54.44±10.11	1.216	0.225
Diabetes course (years)	4.36±3.04	5.27±3.05	1.785	0.075
Smoking history[n%]	20 (28.17)	27 (36.49)	1.144	0.285
BMI (kg/m^2^)	25.54±0.83	27.08±1.49	7.716	<0.001
With hypertension [n%]	15 (21.12)	27 (36.48)	4.155	0.042

**Table II:** Comparison of laboratory indicators between with T2DM and T2DM with NAFLD (*X̅*±*S*).

Laboratory indicators	Simple T2DM (n=71)	T2DM with NAFLD (n=74)	t-test	p-Value
FPG (mmol/L)	8.99±2.09	11.95±3.23	6.562	<0.001
Insulin resistance index	3.28±0.64	4.69±0.91	10.780	<0.001
TC (mmol/L)	4.43±1.05	4.67±1.06	1.377	0.171
TG (mmol/L)	1.42±0.43	1.78±0.30	5.917	<0.001
LDL (mmol/L)	2.71±0.64	2.81±0.73	0.874	0.385
HDL (mmol/L)	1.17±0.27	1.11±0.23	1.463	0.146
HbA1c (%)	8.80±1.32	8.70±1.35	0.442	0.659
ALT (U/L)	21.42±3.46	22.19±3.11	1.413	0.160
AST (U/L)	19.78±2.97	21.44±3.27	3.188	0.002
Cr (μmol/L)	61.55±5.39	60.89±5.11	0.764	0.446
UA (μmol/L)	267.60±31.87	323.35±46.05	8.503	<0.001

**Table III:** Logistic analysis of risk factors for T2DM with NAFLD

Index	B	S.E.	Wald χ^2^	p-Value	OR	95% CI
BMI(kg/m^2^)	0.611	0.247	6.125	0.013	1.841	1.135~2.986
With hypertension(n)	0.254	0.674	0.142	0.706	1.289	0.344~4.833
FPG (mmol/L)	0.455	0.184	6.095	0.014	1.576	1.098~2.262
Insulin resistance index	1.434	0.399	12.891	<0.001	4.195	1.918~9.177
TG(mmol/L)	1.543	0.758	4.143	0.042	4.676	1.059~20.654
AST (U/L)	-0.275	0.149	3.401	0.065	0.759	0.567~1.017
UA (μmol/L)	0.01	0.007	1.87	0.171	1.01	0.996~1.025

***Note:*** B indicates partial regression system; S.E. indicates standard error; Wald χ^2^=(B/S.E.)^2^; OR is odds ratio; 95%CI is the confidence interval of OR.

## DISCUSSION

In this retrospective analysis of T2DM patients with NAFLD, 74 (51.03%) patients were found to be T2DM with NAFLD, suggesting that the incidence rate of NAFLD in T2DM patients was relatively high. Younossi ZM *et al*^13^ in a meta-analysis of 80 studies in 20 countries, 55.5% of 49419 T2DM patients were with NAFLD. It can be seen that the research is consistent with the overall trend. At the same time, logistic regression analysis showed that high levels of BMI, BPG, insulin resistance index, and TG were independent risk factors for NAFLD in T2DM patients.

Obese people have body fat cell volume, which can increase the concentration of insulin receptors on the cell membrane, this can reduce the activation of insulin, and cause insulin resistance, insulin resistance can reduce the rate of glucose uptake and utilization by cells and promote a large amount of free fatty acid intake by liver cells.^14^ This results in an increase in the accumulation of fat within the liver. A recent review by Li L *et al*^15^ observed a significant dose-dependent relationship between BMI and NAFLD risk (for each increase of BMI by 1 unit: RR=1.20, 95% confidence interval 1.14 to 1.26, P<0.001). Specifically, the risk of NAFLD in obese individuals is increased by 3.5 times, and there is a significant dose-dependent relationship between BMI and NAFLD risk. Further, studies have shown that the incidence of fatty liver in people with BMI>24kg/m^2^, BMI>26kg/m2 and BMI>28kg/m^2^ is about 20.7%, 41.9% and 90.0% respectively.^16^ It has been shown that the BMI of patients with T2DM and NAFLD is significantly increased when compared with patients with T2DM alone, which is similar to the results of this study. Therefore, patients with T2DM and NAFLD with high BMI and obesity, should be closely monitored. Intervention measures such as weight control and blood lipid regulation should be initiated to delay the process of further disease advancement.^15^,^16^

Insulin resistance is the basis of T2DM and the main mechanism of development of NAFLD. The liver is the primary organ for the digestion of sugars and lipids.^6^ The decreased fat decomposition in patients with NAFLD can increase gluconeogenesis, resulting in the release and deposition of a large number of visceral adipocytes, promoting the increase in blood glucose.^6^,^7^ Insulin is the main hormone that causes blood glucose to be taken up into the cell.^17^ With T2DM, there is a decrease in the function of pancreatic islets leading to an increase in insulin resistance resulting in an increase in blood glucose.^7^ This causes a further increase in lipid concentration within the liver, contributing to the formation of a fatty liver.^18^ A large number of clinical studies show that insulin resistance is closely related to T2DM complicated with NAFLD.^17^,^18^ Logistic regression analysis confirmed that insulin resistance is the main risk factor of this disease. Therefore, clinical intervention for patients of T2DM with NAFLD should focus on detection and evaluation of islet function. Insulin resistance should be reduced using an insulin sensitizer, so as to improve the prognosis of these patients.^17^

T2DM can cause a large number of free fatty acids to accumulate in the liver, and ultimately transformed into TG.^19^ The oxidation of these free fatty acids in hepatocyte mitochondria can be reduced, which can reduce the secretion of very low-density lipoprotein, resulting in impaired lipid metabolism, hepatocyte damage, and NAFLD.^20^ Recent work has shown that TG levels are significantly increased in patients with NAFLD, suggesting that TG plays an important role in this disease.^21^ As such, when intervening with patients with T2DM, in addition to examining blood glucose concentrations, it is also necessary to measure blood lipid levels, especially TG concentrations.^19^,^20^ If the patient has an abnormal rise in TG, it may be necessary to consider whether the patient has NAFLD as soon as possible and to intervene in time.

In addition, this study also found that there is a positive correlation between AST, ALT, and UA levels with T2DM and NAFLD. AST and ALT are commonly used as clinical indicators of liver function and can reflect the degree of liver injury. UA is the product of purine metabolism and can reflect the degree of insulin resistance and is involved in the occurrence and development of NAFLD through insulin resistance and activation of cytokines.^22^ Although the results of this study showed that the levels of AST, ALT and UA were increased in patients with T2DM and NAFLD, logistic regression analysis did not find those as a risk factor. Studies have shown that age, region, low and high-density lipoprotein, and hypertension are also risk factors for T2DM with NAFLD.^13^,^23^ Although the average age of T2DM with NAFLD in this study was higher than that of simple T2DM, and the high-density lipoprotein was lower than that of simple T2DM, there was no statistical significance, the reason may be that too few samples were included in this study, and patients in other regions were not included, which is also one of the limitations of this study. Some studies have also shown that cardiovascular disease is also a risk factor for NAFLD in T2DM.^24^,^25^ but this time, we have not observed whether the patients have cardiovascular disease, which is also one of the limitations of this article.

### Limitations

The retrospective nature of the study, as well as the small sample size, limited the scope of the overall results.

## CONCLUSION

High BMI, BPG, insulin resistance index and TG are independent risk factors for nonalcoholic fatty liver in T2DM patients. During intervention, attention should be paid to the monitoring of these indicators to effectively prevent the aggravation of the disease.

### Author’s Contributions:

**LC:** Conceived and designed the study.

**LC & LJ:** Collected the data and performed the analysis.

**LC:** Involved in the writing of the manuscript and is responsible for the integrity of the study.

All authors have read and approved the final manuscript.
